# Automated segmentation of multiparametric magnetic resonance images for cerebral AVM radiosurgery planning: a deep learning approach

**DOI:** 10.1038/s41598-021-04466-3

**Published:** 2022-01-17

**Authors:** Aaron B. Simon, Brian Hurt, Roshan Karunamuni, Gwe-Ya Kim, Vitali Moiseenko, Scott Olson, Nikdokht Farid, Albert Hsiao, Jona A. Hattangadi-Gluth

**Affiliations:** 1 Department of Radiation Medicine and Applied Sciences, University of California San Diego, 3960 Health Sciences Dr, Mail Code 0865, La Jolla, CA USA; 2 Department of Radiology, University of California San Diego, La Jolla, CA USA; 3 Division of Neurosurgery, University of California San Diego, La Jolla, CA USA; 4 Department of Radiation Oncology, University of California Irvine, Orange, CA USA

**Keywords:** Medical imaging, Radiotherapy

## Abstract

Stereotactic radiosurgery planning for cerebral arteriovenous malformations (AVM) is complicated by the variability in appearance of an AVM nidus across different imaging modalities. We developed a deep learning approach to automatically segment cerebrovascular-anatomical maps from multiple high-resolution magnetic resonance imaging/angiography (MRI/MRA) sequences in AVM patients, with the goal of facilitating target delineation. Twenty-three AVM patients who were evaluated for radiosurgery and underwent multi-parametric MRI/MRA were included. A hybrid semi-automated and manual approach was used to label MRI/MRAs with arteries, veins, brain parenchyma, cerebral spinal fluid (CSF), and embolized vessels. Next, these labels were used to train a convolutional neural network to perform this task. Imaging from 17 patients (6362 image slices) was used for training, and 6 patients (1224 slices) for validation. Performance was evaluated by Dice Similarity Coefficient (DSC). Classification performance was good for arteries, veins, brain parenchyma, and CSF, with DSCs of 0.86, 0.91, 0.98, and 0.91, respectively in the validation image set. Performance was lower for embolized vessels, with a DSC of 0.75. This demonstrates the proof of principle that accurate, high-resolution cerebrovascular-anatomical maps can be generated from multiparametric MRI/MRA. Clinical validation of their utility in radiosurgery planning is warranted.

## Introduction

Cerebral arteriovenous malformations (AVMs) are congenital cerebrovascular “tangles” of abnormal vessels that shunt blood from arteries to veins. AVMs can be highly morbid, with potential for devastating intracranial hemorrhage^1^. Risk of hemorrhage from an unruptured AVM is reported as high as 2–4% per year, with re-hemorrhage rates approaching 6–7% annually^2–5^. Compounded over a patient’s lifetime, risk of a catastrophic hemorrhage is significant. Stereotactic radiosurgery (SRS) is a standard, non-invasive option for treating cerebral AVMs. Radiosurgery is highly effective for small AVMs (< 10 cc), with obliteration rates as high as 80–90%^6,7^.

However, accurately delineating an AVM nidus for radiosurgical treatment is challenging. Unlike other radiosurgical targets, an AVM nidus is a complex vascular structure that rarely presents as a well-defined mass^8^. Intranidal vessels may be enmeshed in normal brain tissue, extranidal vessels may surround the nidus, and in the post-embolization setting, portions of nidus are filled with embolic agents^9^. The visual appearance of intranidal arteries, which are the radiosurgical target, relative to the surrounding intracranial tissue, is highly dependent on the choice of imaging modality^9–11^, and under-coverage of the nidus can lead to treatment failure^7^. Compounding this challenge, AVM radiosurgery can cause high-grade toxicity in some cases, with dosimetric studies showing an association between the volume of intracranial tissue receiving a high dose of radiation and the risk of treatment-related adverse events, including radionecrosis^12,13^. Radiosurgical planning often requires tradeoffs between obliteration likelihood and toxicity risk, and great care must be taken to ensure that the nidus is fully treated while minimizing the overall treatment volume. As such, even with advanced imaging, contouring AVMs for radiosurgery requires neurovascular expertise and reference to multiple imaging studies.

With the goal of facilitating nidus delineation, we developed a formal method of combining the vascular anatomical information contained in multiple complementary high-resolution magnetic resonance imaging (MRI) sequences into a single three dimensional (3D) synthetic image volume representing the relevant anatomical structures for AVM radiosurgery treatment planning. The synthetic image volume delineates potential target tissue (arteries) from tissue at risk (brain parenchyma) and from other relevant tissue classes (veins, cerebral spinal fluid, embolized vessels) at a voxel scale and represents a high resolution vascular anatomical map of the whole intracranial volume. This type of image segmentation is highly suited to automation by a machine-learning derived algorithm, and we selected a convolutional neural network (CNN) for this task given its ready application to imaging datasets. Note that while CNNs typically require several hundred to several thousand training examples to produce reasonable predictions, AVMs are relatively rare, and patient databases are often an order of magnitude smaller. Fortunately, MR image volumes contain 100 s of slices, each of which contains multiple tissue classes relevant to training. Thus, by employing a two-dimensional (2D) CNN, thousands of training and validation examples can be generated from a data set containing a few dozen patients.

## Results

### Patient characteristics

**Figure 1:**
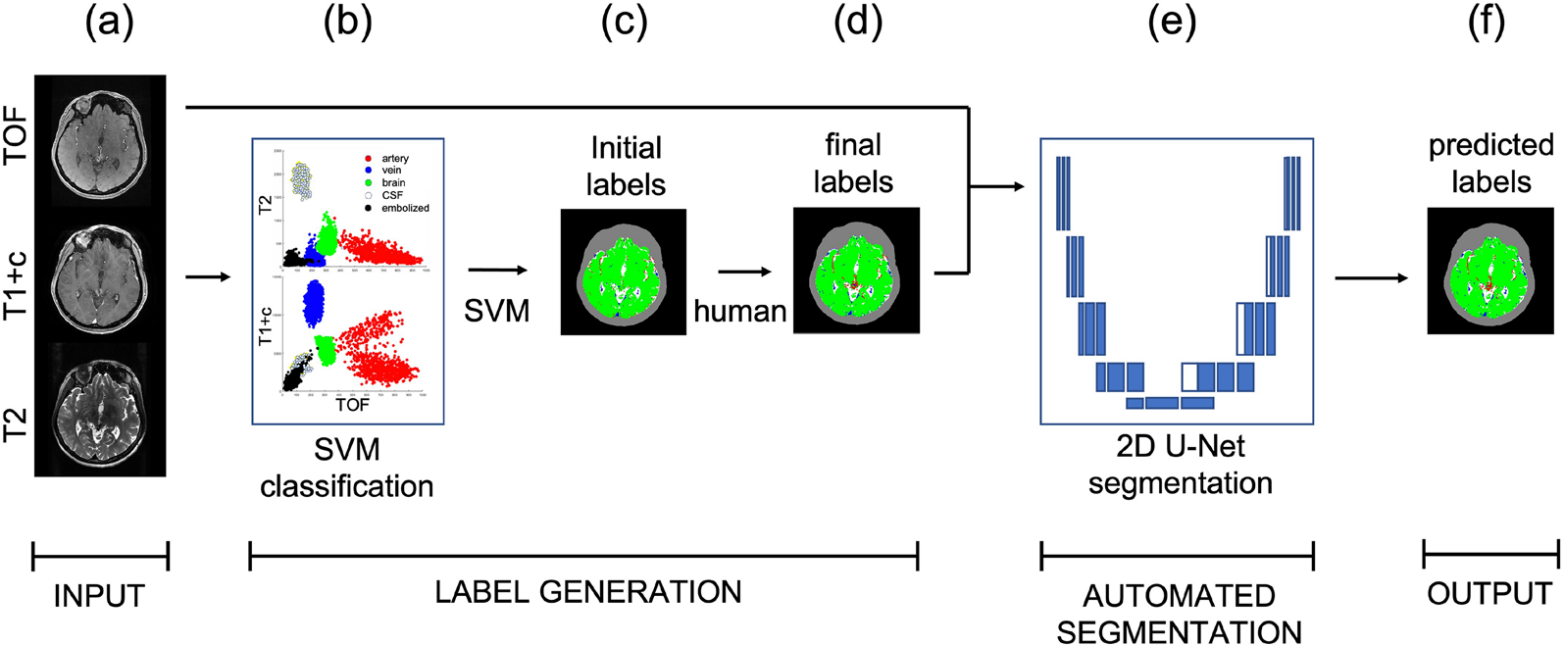
Workflow schematic for algorithm development. (**a**) Magnetic resonance images are co-registered. (**b**) Labels are generated using a support vector machine-based (SVM) algorithm to classify voxels into arteries (red), veins (blue), brain (green), CSF (white), and embolized vessels (black). (**c**,**d**) Labels are edited manually. (**e**) Final labels and co-registered images are used to train and validate the convolutional neural network. (**f**) Output is a 3D map of the predicted voxel labels. T1 + c: T1 post-contrast; TOF: time-of-flight. CSF: cerebral spinal fluid.

Twenty-three patients were available for analysis. Eleven patients in the training cohort (65%) and 4 patients in the validation cohort (66%) had undergone partial embolization. Nine patients in the training cohort (53%) and 4 patients in the validation cohort (66%) had experienced a prior bleed (Fig. 1).

### Model performance

In the training data set, 0.9%, 4.2%, 84.3%, 10.4%, and 0.2% of voxels were labeled as arteries, veins, brain parenchyma, cerebral spinal fluid (CSF), and embolized vessels (EV), respectively. In the validation set, 1.3%, 6.9%, 80.1%, 11.6%, and 0.1% of voxels were labeled as belonging to arteries, veins, brain parenchyma, CSF, and embolized vessels, respectively. Once training was complete, processing time for model predictions was approximately 49 ms per slice, or 60 s for the entire validation training set. Model performance was high across each of the classification labels in the training data set, with Dice Similarity Coefficients (DSC) of 0.90, 0.94, 0.99,0.97, and 0.93 for arteries, veins, brain parenchyma, CSF, and embolized vessels, respectively. In the validation data set, performance remained high for arteries, veins, brain parenchyma, and CSF with DSCs of 0.86, 0.91, 0.98, and 0.91, respectively (Table 1). Performance was lower for embolized vessels in the validation data set, with DSC of 0.75.

**Table 1:** Dice similarity coefficients for each tissue class and for each subject in the validation data set.

	Arteries	Veins	CSF	Brain	Embolized
Subject 1	0.88	0.93	0.92	0.98	0.69
Subject 2	0.87	0.87	0.91	0.98	0.74
Subject 3	0.82	0.93	0.89	0.98	N/A
Subject 4	0.91	0.90	0.93	0.99	N/A
Subject 5	0.86	0.84	0.93	0.98	0.52
Subject 6	0.87	0.95	0.89	0.98	0.84
Combined dataset	0.86	0.91	0.91	0.98	0.75

**Figure 2:**
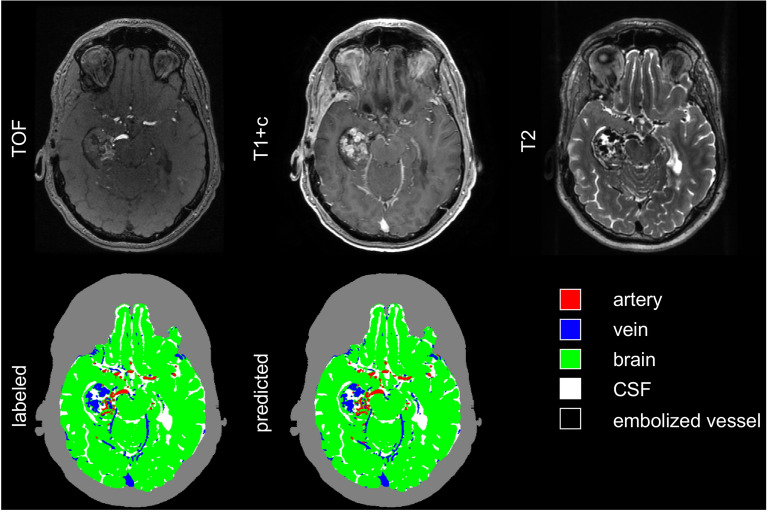
Representative images of an AVM nidus in the right temporal lobe as demonstrated by multi-modal MRI/MRA and vascular anatomical maps. Dice similarity coefficients for this slice: arteries 0.85; veins 0.84; brain 0.98; CSF 0.94; embolized vessels 0 (not present). The grey contour outlining the extracranial space is not generated by the segmentation algorithm and is included for visual reference only. AVM: arteriovenous malformation, MRI: magnetic resonance imaging, MRA: magnetic resonance angiography, CSF: cerebral spinal fluid.

**Figure 3:**
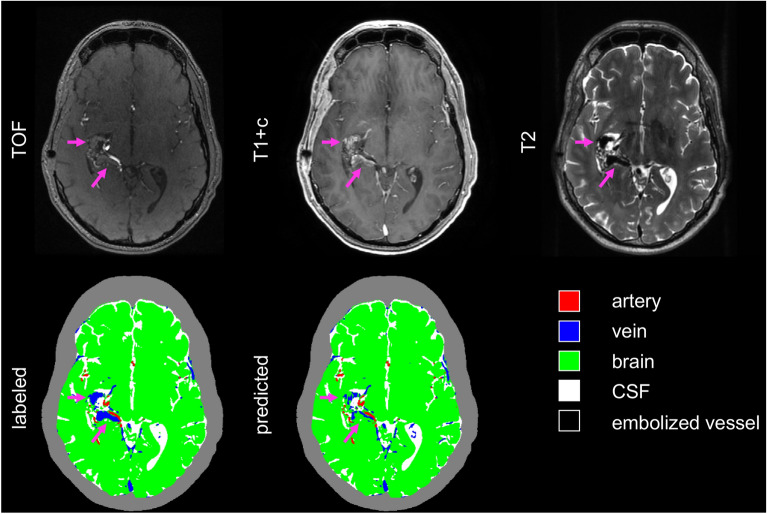
Representative images demonstrating misclassification within a poorly enhancing draining vein. Purple arrows show the location of the draining vein, which demonstrates flow-voids on T2 but does not avidly enhance on T1 + c images. The neural network correctly classifies portions of the vessel where contrast enhancement is present but misclassifies non-enhancing voxels as brain parenchyma. Dice similarity coefficients for this slice are as follows: arteries 0.88; veins 0.73; brain 0.98; CSF 0.92; embolized vessels 0 (not present). The grey contour outlining the extracranial space is not generated by the segmentation algorithm and is included for visual reference only. T1 + c: T1 post-contrast; TOF: time-of-flight. CSF: cerebral spinal fluid.

**Figure 4:**
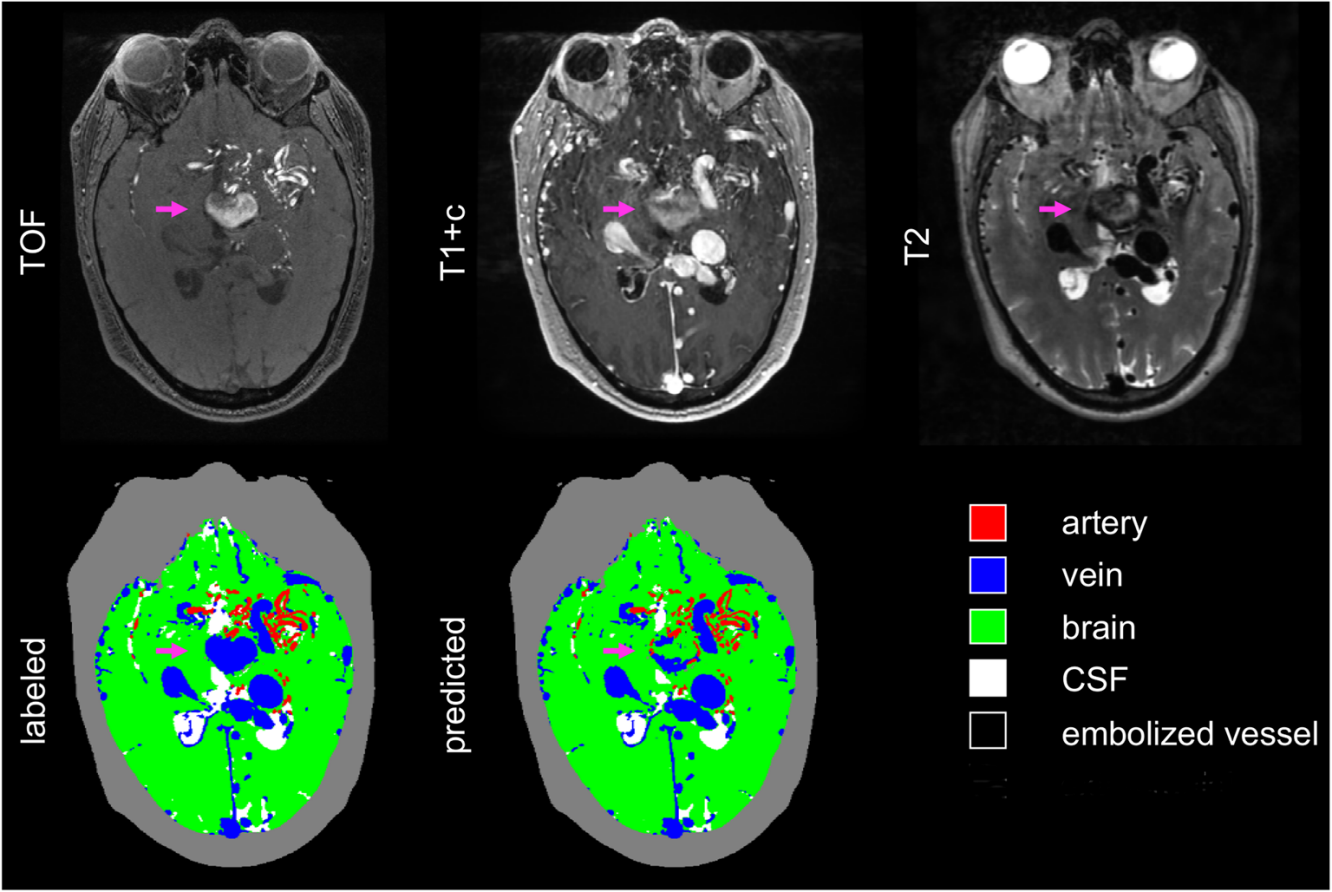
Representative images demonstrating misclassification within a thrombosed draining vein. Purple arrows show the location of the thrombosed vein which lacks characteristic flow-voids on T2, enhances poorly on T1 + c, and is bright on TOF. The neural network misclassifies portions of this vessel as brain parenchyma or artery. Dice similarity coefficients for this slice are as follows: arteries 0.86; veins 0.9; brain 0.96; CSF 0.74; embolized vessels 0 (not present). The grey contour outlining the extracranial space is not generated by the segmentation algorithm and is included for visual reference only. T1 + c: T1 post-contrast; TOF: time-of-flight. CSF: cerebral spinal fluid.

**Figure 5:**
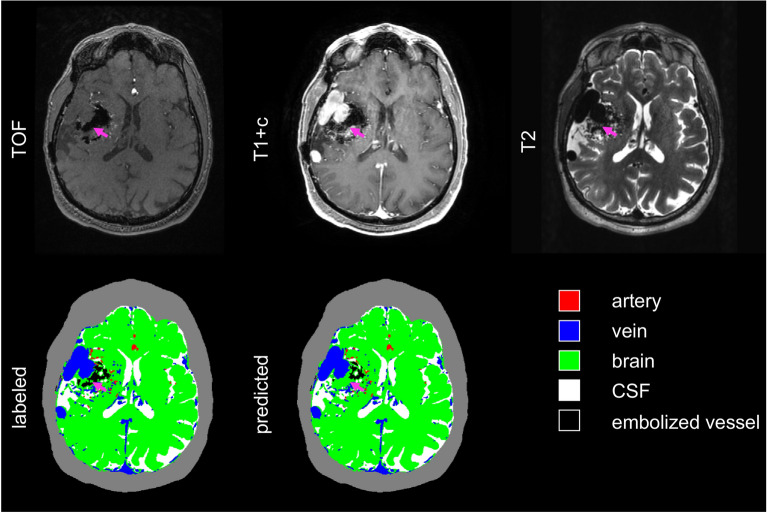
Representative images showing under-classification of embolized vessels within in a complex, partially embolized nidus. Purple arrows show the embolized vessels. Dice similarity coefficients for this slice are as follows: arteries 0.81; veins 0.95; brain 0.98; CSF 0.95; embolized vessels 0.64. The grey contour outlining the extracranial space is not generated by the segmentation algorithm and is included for visual reference only. T1 + c: T1 post-contrast; TOF: time-of-flight. CSF: cerebral spinal fluid.

Figure 2 shows a representative axial image slice through the AVM nidus for a case in the validation set, demonstrating visually good agreement between labeled and predicted classifications, with DSCs for this slice comparable to the dataset as a whole. The full image volumes for each case in the validation set can be viewed in video format in the supplemental material (Supplemental Videos 1–6). Of note, there were several distinct imaging-anatomical features which the model did not correctly classify. Examples of these included a large draining vein which was not strongly enhancing on T1-post contrast imaging (Fig. 3) and a large, thrombosed vein which was hyperintense on TOF, despite absence of associated T2 flow void or contrast enhancement (Fig. 4). The model also tended to under-predict embolized vessels (Fig. 5), possibly due to the relatively low number of voxels representing embolized vessels in the training dataset.

**Figure 6:**
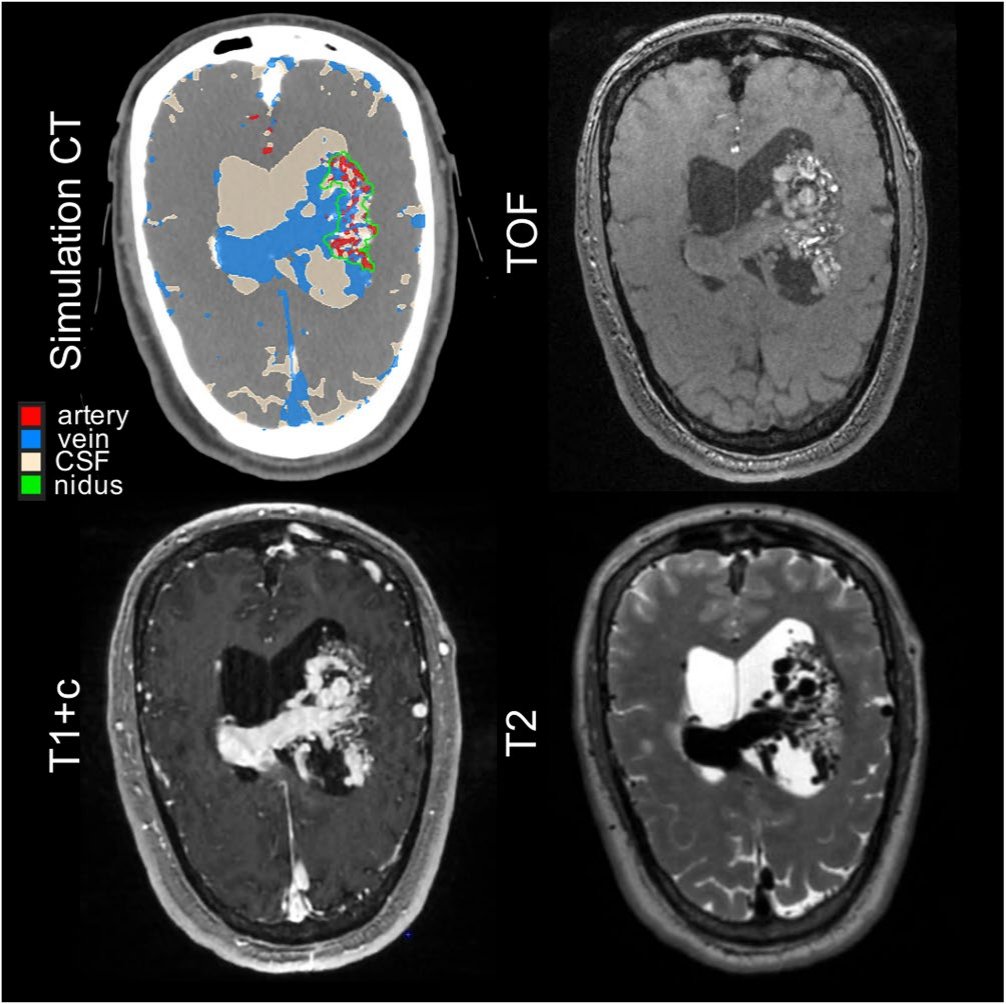
Application to radiosurgery treatment planning. Predicted maps have been loaded into treatment planning software, converted into contours, and overlaid onto simulation CT, where they can provide additional visual context for contouring. T1 + c: T1 post-contrast; TOF: time-of-flight. CSF: cerebral spinal fluid. CT: computed tomography.

Figure 6 shows potential application of vascular-anatomical mapping to target delineation. The patient has a large AVM wrapped around a network of dilated, deep draining veins. Predicted maps were uploaded to treatment planning software, registered with the treatment planning CT, converted into contours, and overlaid with the simulation CT (top left). Overlaid maps are then viewable simultaneously with raw MRI/MRA sequences from which they are derived. Note that the boundary of the AVM is difficult to discern in any of the individual MR sequences individually but is more clearly visible in the overlaid map. The contoured boundary of the AVM nidus is shown in green.

## Discussion

Radiosurgical management of cerebral AVMs is challenged by limitations of target visualization, with nidus appearance heavily dependent on the chosen imaging modality. Here, we demonstrated the proof of principle that the vascular anatomical information contained in multiple standard clinical MR image sequences can be accurately combined into a single high-resolution vascular anatomical map of the whole brain with automatic segmentation of the relevant structures for radiosurgery planning.

Despite a relatively small cohort size, we achieved DSCs of 0.86, 0.91, 0.98, 0.91, and 0.75 for arteries, veins, brain parenchyma, CSF, and embolized vessels, respectively, across an anatomically variable validation dataset. These values are considered good–excellent in the image segmentation literature^14,15^. The vascular-anatomical map produced by our algorithm represents an easily visualizable synthesis of three complementary MR sequences and may provide useful visual context for delineating complex lesions. Future work to validate this will include a study of the impact of this tool on inter-practitioner agreement in target delineation.

This work is not the first effort to apply machine learning to the delineation of the AVM nidus. Peng et al. applied fuzzy c-means clustering to T2 weighted MR images used for gamma-knife treatment planning to segment CSF, brain-parenchyma, and vessels within the prescription isodose volume^16^. Limitations of that work included use of a single T2-weighted MRI sequence that cannot distinguish arteries from veins and applicability to only a small volume of interest within the brain. Wang et al. developed a deep learning approach to automated contouring of an AVM nidus based on contrast computed tomography (CT) images^17^. Within their dataset they achieved a high level of agreement between automatically and manually contoured lesions. While that approach is also promising, limitations include the need to crop the input image volume into a 64 × 64x64 voxel volume of interest. Further, the algorithm’s reliance on contrast CT imaging alone as an input may limit the types of lesions to which this algorithm may be applicable. Draining veins, calcifications, blood products, and embolization fluid can appear similarly to contrast enhanced intranidal arteries on CT, and the latter can create profound artifacts on CT images. Potential advantages of our approach over these methods include its applicability to whole brain volumes and employment of multiple MR image sequences, which increase its ability to discriminate arterial vessels from other intracranial tissues and vascular structures. A potential advantage of tissue segmentation over direct nidus delineation includes the ability to derive tissue-specific dose volume histograms from the output of the segmentation algorithm^18^, not just for the nidus itself, but for normal tissues inside and outside of the target volume. In addition, for complex lesions such as those that have been partially embolized, contain large draining veins, or are of the diffuse type with admixed vessels and brain parenchyma, there is potential for subjectivity in terms how the target volume is delineated. Anatomical maps can aid in complex decision making by clarifying the relevant anatomy without limiting physician autonomy. Finally, these vascular maps provide an entire whole-brain picture of vascular relationships within and around the nidus, including collateral vessels. Such vascular maps will allow for a better understanding of treatment response, for example changes in draining veins which often can precede changes in the nidus volume itself after SRS.

Outside of the radiosurgerical literature, several groups have recently published strategies for automatically generating vascular maps for potential use in analysis of a range of cerebral vascular diseases. For example, Avadiappan et al. utilized an adaptive Frangi filter to segment arterial vessels and estimate vessel radii from MRA image volumes, finding DSCs ranging from 0.83 to 0.89 in comparison with manually segmented vessels^19^. Similarly, Hilbert et al. developed a 3D convolutional neural network named BRAVE-NET to segment arterial vessels based on MRA, finding an average DSC of 0.931 on an internal test dataset and 0.746 on an external validation dataset^20^. Meijs et al. also employed a 3D convolutional neural network to automatically segment arteries and veins from 4D CT angiograms, finding a mean DSC of 0.8 for arteries and 0.88 for veins in their test data set^21^. It is difficult to directly compare the performance of our algorithm with these and other automated vascular segmentation algorithms given the potential effects of differences in subject population, imaging hardware and techniques employed, manual contouring technique, and random variation, however, it is encouraging to note that our results for arterial and venous segmentation are comparable to other algorithms designed to achieve similar, if not identical goals. Advantages of our approach for use in radiosurgery planning include the ability to simultaneously segment potential target structures (arteries), organs at risk (brain), and structures that may neither require deliberate coverage nor avoidance (veins, CSF, embolized vessels). In addition, as noted above, CT angiography may be of limited utility in the post-embolization setting due to embolisate-produced artifacts.

This study has several limitations. While considerable attention was paid in voxel-wise labeling of training and validation datasets, given the high-resolution images, small variations in image contrast and quality, and large number of voxels, there may be voxels within the training and validation datasets that are mislabeled. While the overall visual appearance of the labels is good, there may be regions within a compact nidus, for example, where small arteries are labeled as veins and vice-versa. We believe that most of such errors are likely to be random in nature, and thus, while they may impose an upper ceiling on algorithm performance, they are less likely to bias the algorithm in a clinically significant way. Second, as our training cohort was relatively small, the model’s performance was more limited with low-prevalence anatomical features (e.g. thrombosed and embolized vessels). Further performance improvements are achievable by increasing the number of cases containing these features in the training dataset. Other approaches to improving performance, such as employing an alternative loss function or alternative network architecture, are also worth exploring as we obtain additional data. Third, while the Dice Coefficient, which was utilized here to evaluate performance, is amongst the most commonly utilized metrics in the image segmentation literature, it is understood that segmentation algorithms that achieve high Dice scores do not uniformly demonstrate clinical utility^22^. Further, the Dice Coefficient itself possesses certain limitations, including an inability to distinguish systematic from random errors and a tendency to provide higher scores to structures with larger volumes^23^. Fourth, this algorithm does not automatically segment the intracranial compartment as a whole and will generate labels for structures outside of the calvarium. Thus to use this algorithm clinically, an externally generated inner calvarium contour must be utilized. In practical terms, this does not present a significant impediment to utilization or workflow as most standard clinical treatment planning programs already contain highly accurate routines for segmenting the intracranial volume. Finally, all imaging studies were performed at a single institution. It is thus uncertain whether performance would be maintained with similar, but not identical, MR sequences. A goal for future work is to collaborate with other institutions to assess the algorithm’s performance with other imaging platforms and employ transfer learning to increase its compatibility.

In conclusion, we have demonstrated a proof of principle for automatic synthesis of vascular-anatomical maps from multi-modal MRI/MRA for patients with cerebral arteriovenous malformations. The algorithm demonstrated good discriminatory performance across an anatomically highly diverse set of validation images. This work represents *the first effort to generate high-resolution, whole-brain vascular-anatomical maps for AVM patients*. Future work will include further improvements in performance through training on larger datasets and formal evaluation of its utility in radiosurgery treatment planning.

## Methods

### Human subjects

Study subjects were enrolled on a single-institution prospective study evaluating the utility of a novel MRI/MRA sequence in patients with cerebral AVMs. The study was approved by the UC San Diego Institutional Review Board (IRB# 170848), and informed consent was obtained from all subjects or their legally authorized representatives. All methods were carried out in accordance with institutional guidelines and regulations. All patients were either evaluated for treatment with SRS or had undergone SRS previously.

### Imaging

Imaging sequences chosen as inputs included: 3D time of flight (TOF) MRA, 3D T1-weighted post contrast (T1 + c) MRI, and 3D T2-weighted (T2) MRI. These were selected because they provide complementary information about vascular anatomy of the AVM nidus and surrounding structures (Table 2), are acquired with high-resolution 3D acquisition sequences with sub-millimeter in-plane resolution and millimeter through-plane resolution, and are used clinically at our institution for AVM target delineation. Images were acquired on a GE Medical Systems Discovery MR750 3T magnet. T1-post contrast images were acquired with a 3D FSPGR sequence after injection of Gadobenate contrast. Time-of-flight (TOF) MRA was based on a 3D non-contrast SPGR sequence. T2 images were based on a 3D spin-echo sequence. Images were reconstructed axially with in-plane matrix of 512 × 512, in plane field of view of 220-250 mm, and slice thickness of 1–1.4 mm. T1 + c and T2 image volumes covered the whole head. For TOF MRA, a slab of variable thickness was acquired that encompassed the AVM nidus and most of the brain.

**Table 2:** Typical relative voxel intensities for each histological class for each of the MR sequences used in the study. ^*^Early draining veins can have high intensity signal on TOF. ^+^arterial intensity can be variable on T1 + c.

	Arteries	Veins	CSF	Brain	Embolized
TOF	High	Moderate*	Low-moderate	Moderate	Low
T1 + c	High^+^	High	Low-moderate	Moderate	Low
T2	Low	Low	High	Moderate	Low

### Image preprocessing

For each case, Digital Imaging and Communications in Medicine (DICOM) image files were imported into standard radiosurgery treatment planning software. T1 + c and T2 images were rigidly registered to the TOF images and visually inspected for quality. Registered T1 + c and T2 images were resampled to match the spatial resolution, slice number, and field of view of TOF images (Fig. 1a).

### Image Labeling:

Voxel labels were generated with hybrid semi-automated and manual techniques. First, an intracranial contour was generated using semi-automated tools included in the treatment planning software. Next, for each case, a sample of each intracranial voxel label (arteries, veins, brain parenchyma, CSF, and EV (when present)) was contoured. Registered images and contours were then exported into MATLAB (v.2017b, Natick, MA). Voxel intensity values for each image sequence within each of the sample contours were extracted. These values were then used to train a machine learning algorithm (*fitcecoc* in MATLAB^24,25^) for multiclass prediction based on support-vector-machines and error-correcting output codes (Fig. 1b). The trained model was then used to predict the class (artery, vein, brain parenchyma, CSF, EV) of each intracranial voxel. This model was trained for each subject from sample contours. Its output was a rough vascular-anatomical map of the whole brain (Fig. 1c).

Next, image label volumes were reviewed and meticulously edited by a radiation oncologist with neurovascular expertise and experience treating AVMs. The final output of this process was a 4D array (row, column, slice, label) of classification labels for each image volume, where each voxel was labeled as belonging exclusively to artery, vein, brain parenchyma, CSF, or EV (designated 1 if belonging or 0 if not) (Fig. 1d). Extracranial voxels were unlabeled and excluded from training or validation. Note that for one subject in the validation set, an implanted intracranial device created a large imaging artifact in the brain well outside of the region of the AVM nidus (Supplemental Video 6). Because of the severity of this artifact which made voxel labeling infeasible, the affected region was excluded from analysis (labeled as extracranial).

### Training the convolutional neural network

Labeled maps and images from the training dataset were loaded into the CNN model (Fig. 1e), which was implemented in Python 3.7.3 using the Keras Deep Learning Library and running TensorFlow backend. Image intensities were normalized from 0–1 on a per-slice basis using simple linear normalization. Images from 17 patients (3181 image slices) were used for training. Patients were allocated randomly to training or validation sets with an effort to balance the proportion of subjects who had undergone embolization in each group. The training set was augmented by left to right reflection, generating a total of 6362 training slices. The architecture of the CNN was based on the 2D U-Net^26^. Minor differences between this network and the original U-net were as follows: the number of input image channels was increased from one to three to accommodate the 3 MRI contrasts; images were initially zero-padded to a matrix size of 632 × 632 in the axial plane to accommodate image cropping from convolution and produce an output image with the initial matrix size (512 × 512); the final layer was a 1 × 1 convolution with 5 rather than 2 output channels, to accommodate the 5 classification categories. As in original U-Net, a voxel-wise softmax function was used for the final activation function. A categorical cross-entropy function was used as a loss function. The Adam algorithm was used for gradient-descent optimization. A batch size of 6 was used for training. Twenty training epochs were used.

### Evaluation of model performance

Prediction performance was evaluated for 6 subjects (1224 image slices) in the validation data set. As the output of the model is non-binary, the single classification category with the maximum prediction score was assigned to each output voxel (Fig. 1f.). No attempt to optimize the threshold for classification was made as there was insufficient data to create an additional testing dataset for hyperparameter tuning. After assigning a label to each voxel, predicted maps were compared to labeled maps in a category-by-category fashion, using Dice Similarity Coefficient (DSC) as a metric for performance. DSC was defined as

$${DSC}_{x}= \frac{2|{X}_{label}\cap {X}_{pred}|}{\left|{X}_{label}\right|+|{X}_{pred}|}$$

where *DSC*_*x*_ is the coefficient for category x (e.g. artery, vein), $\left|{X}_{label}\right|$ is the number of voxels labeled *x*, $\left|{X}_{pred}\right|$ is the number of intracranial voxels predicted as *x*, and $|{X}_{label}\cap {X}_{pred}|$ is the number of voxels labeled *and* predicted as *x.*

## Supplementary Information

Supplementary Video Legends.

Supplementary Video 1.

Supplementary Video 2.

Supplementary Video 3.

Supplementary Video 4.

Supplementary Video 5.

Supplementary Video 6.

## Data Availability

Research data are stored in an institutional repository and will be shared upon request to the corresponding author.
