# Supplementary material for: Automated segmentation of multiparametric magnetic resonance images for cerebral AVM radiosurgery planning: a deep learning approach
Source: Sci Rep. 2022 Jan 17;12:786. doi: 10.1038/s41598-021-04466-3 (PMC8763944; doi:10.1038/s41598-021-04466-3)
Supplement: Supplementary file 1 — Supplementary Video Legends. [file 41598_2021_4466_MOESM1_ESM.docx]

**Supplemental Video Legends**

Supplemental Video 1: Cerebrovascular-anatomical map of a patient with a large right frontal AVM who had undergone prior partial embolization. The top row shows the input MR imaging volumes, which from left-to-right include time-of-flight (TOF), contrast enhanced T1-weighted (T1+c), and T2-weighted (T2) sequences. The bottom row shows the manually labeled map (left) and the map predicted by the convolutional neural network (middle). The legend designates the label assigned to each voxel in the volume. The grey contour outlining the extracranial space is not generated by the segmentation algorithm and is included for visual reference only.

Supplemental Video 2: Cerebrovascular-anatomical map of a patient with a large, central AVM who had undergone prior partial embolization. The top row shows the input MR imaging volumes, which from left-to-right include time-of-flight (TOF), contrast enhanced T1-weighted (T1+c), and T2-weighted (T2) sequences. The bottom row shows the manually labeled map (left) and the map predicted by the convolutional neural network (middle). The legend designates the label assigned to each voxel in the volume. The grey contour outlining the extracranial space is not generated by the segmentation algorithm and is included for visual reference only.

Supplemental Video 3: Cerebrovascular-anatomical map of a patient with a large AVM in the left lateral ventricle with a thrombosed draining vein. The top row shows the input MR imaging volumes, which from left-to-right include time-of-flight (TOF), contrast enhanced T1-weighted (T1+c), and T2-weighted (T2) sequences. The bottom row shows the manually labeled map (left) and the map predicted by the convolutional neural network (middle). The legend designates the label assigned to each voxel in the volume. The grey contour outlining the extracranial space is not generated by the segmentation algorithm and is included for visual reference only.

Supplemental Video 4: Cerebrovascular-anatomical map of a patient with a small left temporal lesion that was later favored to represent a developmental venous anomaly. The top row shows the input MR imaging volumes, which from left-to-right include time-of-flight (TOF), contrast enhanced T1-weighted (T1+c), and T2-weighted (T2) sequences. The bottom row shows the manually labeled map (left) and the map predicted by the convolutional neural network (middle). The legend designates the label assigned to each voxel in the volume. The grey contour outlining the extracranial space is not generated by the segmentation algorithm and is included for visual reference only.

Supplemental Video 5: Cerebrovascular-anatomical map of a patient with a right temporal AVM who had undergone prior partial embolization. The top row shows the input MR imaging volumes, which from left-to-right include time-of-flight (TOF), contrast enhanced T1-weighted (T1+c), and T2-weighted (T2) sequences. The bottom row shows the manually labeled map. (left) and the map predicted by the convolutional neural network (middle). The legend designates the label assigned to each voxel in the volume. The grey contour outlining the extracranial space is not generated by the segmentation algorithm and is included for visual reference only.

Supplemental Video 6: Cerebrovascular-anatomical map of a patient with a right temporal AVM who had undergone prior partial embolization. The top row shows the input MR imaging volumes, which from left-to-right include time-of-flight (TOF), contrast enhanced T1-weighted (T1+c), and T2-weighted (T2) sequences. The bottom row shows the manually labeled map (left) and the map predicted by the convolutional neural network (middle). The legend designates the label assigned to each voxel in the volume. The grey contour outlining the extracranial space is not generated by the segmentation algorithm and is included for visual reference only. Note that an implanted intracranial device produced a large artifact across multiple sequences in the left cerebral hemisphere. Where artifact precluded accurate labeling of the brain, voxels were excluded from the performance analysis.
